# Genome-wide association study of blood vitamin D metabolites and bone remodelling markers in pigs

**DOI:** 10.1186/s12864-025-11914-1

**Published:** 2025-08-02

**Authors:** Dipanwita Paul, Michael Oster, Siriluck Ponsuksili, Klaus Wimmers, Henry Reyer

**Affiliations:** 1 Research Institute for Farm Animal Biology (FBN), Wilhelm-Stahl-Allee 2, 18196 Dummerstorf, Germany; 2 Faculty of Agricultural, Civil and Environmental Engineering, University Rostock, Justus-von-Liebig-Weg 6, 18059 Rostock, Germany

**Keywords:** Bone development, Genetics, Genomic heritability, Micronutrient utilization, Musculoskeletal system

## Abstract

**Background:**

Bone integrity is crucial for farm animals, particularly pigs, as it has direct impact on animal health and welfare as well as for a sustainable livestock farming. Blood serum provides valuable insight into bone metabolism and turnover by assessing key indicators of mineral utilization and bone development, such as serum calcidiol (vitamin D_3_ storage form), calcitriol (vitamin D_3_ active form), β-CTX (C-terminal telopeptide), and CICP (type I C-terminal collagen propeptide). Due to the substantial inter-individual variation observed in serum levels of vitamin D metabolites and bone remodelling markers, this study aimed to investigate the genetic contributions to this variability. The genetic determinants of serum calcidiol, calcitriol, β-CTX, and CICP were investigated in a population of 610 purebred German Landrace pigs. Genetic diversity was maximized by selecting individuals (sib-pairs) from different litters, with animals aged 166 ± 14 days. The phenotypic traits were investigated by a genome-wide association study (GWAS).

**Results:**

Genomic heritability estimates for these phenotypes showed moderate to low heritability with serum calcidiol (0.14), calcitriol (0.12), ß-CTX (0.15), and CICP (0.12). A total of 17 genomic regions on different chromosomes were identified that contain single-nucleotide polymorphisms significantly associated with these four phenotypes. The integration of positional and functional aspects revealed a total of 23 candidate genes with the highest relevance of *PTH*, *GC*, *ALB* for serum calcidiol, *PDPN* for serum calcitriol and *BTG1*, *FASN*, *FOXK2* for serum ß-CTX, and *RETSAT*, *ATOH8*, *FGF11*, *ALOX15* for serum CICP.

**Conclusion:**

The results emphasize the potential for developing innovative breeding criteria that specifically target bone health in pigs. Hence, mineral utilization efficiency might be indirectly improved which remains to be empirically demonstrated through further research.

**Supplementary Information:**

The online version contains supplementary material available at 10.1186/s12864-025-11914-1.

## Background

Pigs achieve calcium (Ca) and phosphorus (P) homeostasis through complex physiological mechanisms [1, 2]. Physiological Ca is essential for blood clotting, muscle contraction, and enzymatic activities, while P is crucial for nucleic acid synthesis, ATP production, blood pH buffering, and membrane fluidity. Moreover, these minerals form hydroxyapatite in bones and teeth, with an optimal Ca/P ratio of 1.50 to 1.66, necessary for effective bone mineralization [1, 3]. This delicate mineral balance, collectively referred to as mineral homeostasis, is maintained through a complex endocrine network that includes vitamin D metabolites (i.e., calcidiol and calcitriol), which interact with their respective receptors [1, 4–6]. Calcitriol, the active form of vitamin D, is produced from its storage form calcidiol through sequential hydroxylation in e.g. the liver (CYP2R1, CYP27A1) and kidneys (CYP27B1) to promote intestinal mineral absorption and bone mineralization [7, 8]. For optimal bone integrity, key components of the organic matrix, primarily collagens, serve as a structural scaffold continuously deposited by osteoblasts and mobilized by osteoclasts in a tightly regulated, dynamic process [9]. In fact, bone remodeling is expressed by bone formation markers such as type I C-terminal collagen propeptide (CICP) and bone resorption markers such as C-terminal telopeptide (ß-CTX) in serum [10, 11].

As reported previously, genetics influence serum calcidiol and calcitriol levels in humans [12, 13]. Nucleotide polymorphisms in candidate genes such as *CYP2R1* and *GC* are known to impact on circulating vitamin D metabolites in humans [14, 15]. Moreover, a few variants in key factors such as *SLC34A1*, *CASR*, *FGF23*, and *TRAFD1* have been described as contributing to the variability in mineral homeostasis [16–20]. In pigs, these functional candidate genes (*SLC34A1*, *FGF23*, *and TRAFD1)* were also found to be associated with serum mineral levels [21]. A genome-wide association study (GWAS) revealed candidate genes including *DDX42*, *MYSM1*, and *FTSJ3* to be associated with bone mineral density (BMD) in Yorkshire pigs [22]. A recent study reported that BMD is moderately heritable (0.21–0.31) and two quantitative trait loci (QTL) on chromosome 6 containing three candidate genes, i.e., *Lin28a*, *CNR2*, and *ZBTB40*, were highlighted as putative key regulators of porcine BMD [23].

In the literature, heritability estimates for circulating calcidiol levels in humans vary widely from 0.075 to 0.16 and 0.70, indicating that rare variants and gene-environment interactions remain underexplored [16, 24, 25]. Moreover, the heritability estimates for mineral balance represented by serum Ca and inorganic P levels are 0.33 and 0.58, respectively in humans [26]. A GWAS involving 1053 German Landrace pigs reported a considerable genetic contribution to the variability of blood Ca, blood P, the respective Ca/P ratio, and alkaline phosphatase activity (ALP). Specifically, the genomic heritability estimates suggested that up to 42% of the variation in blood P levels and 27% in blood Ca levels is attributed to genetics [20] which corresponds well with reports of other species including humans. Farm animal husbandry controls dietary mineral intake, environmental conditions, and parentage [27], and therefore provides a valuable opportunity to dissect the genetic architecture of the vitamin D system and advance the understanding of mineral homeostasis and bone health in mammals.

The hypothesis of the study is that the variations in porcine serum calcidiol and calcitriol levels and proxies for bone mineralization are due to genetic differences leading to specific patterns of hub genes. This knowledge can ultimately be applied to develop methods for selection and mating decisions. This study aims to elucidate the genetic factors influencing individual variability in mineral utilization by focusing on the vitamin D system and bone remodelling markers in a population of purebred German Landrace pigs.

## Material and methods

### Pig population

This study utilized data collected over a 10-year period from a semi-commercial pig production under controlled housing conditions and standardized diets. From the overall resource population of  > 4000 pigs a total of 610 institutional purebred German Landrace pigs were selected. The primary selection criteria involved sex (castrated males and females), unique parentage (maximum of 2 full-sibs), uniformity in slaughter weight, and age. The selected pig population comprised 178 females and 432 castrated males originating from 86 boars and 458 sows. Animals had an average age of 166 ± 14 days (mean ± SD) and an average body weight of 116.5 ± 5.1 kg. Animals were raised on commercial soy/corn based standard fattening diets with ad libitum access to feed and water. The total dietary Ca and P contents were 0.65% and 0.50%, respectively. Diets were supplemented with 265 FTU/kg 6-phytase in line with nutritional guidelines for fattening pigs in the mid-to-late growth phase. The pigs were fasted for 4 h and then euthanized by electrical stunning followed by exsanguination in the experimental slaughterhouse of the FBN Dummerstorf. Blood was drawn from the cranial vena cava at slaughter, centrifuged (3000 RCF, 4 °C), and the resulting serum stored at − 80 °C. Liver samples were collected and stored at − 80 °C until further use.

### Phenotypic analysis of vitamin D metabolites and bone remodelling markers

Serum calcidiol (EIA-5396, DRG, Marburg, Germany), calcitriol (AC-62F1, Immunodiagnostic Systems GmbH, Frankfurt am Main, Germany), β-CTX (AC-02F1, Immunodiagnostic Systems GmbH) and CICP (MicroVue Bone, Quidel, Athens, OH, USA) were measured using commercially available ELISA kits according to the manufacturer’s instructions in duplicates. Metabolite concentrations were determined from a standard curve of serial dilutions using four-parameter logistic curve fits in Mars-Omega data analysis software (BMG Labtech, Germany, v4.01.R2). Data preprocessing and analysis were performed in R (v4.1.1; R Foundation for Statistical Computing, Vienna, Austria). Data distributions were assessed for normality using the Shapiro–Wilk test (stats package), and Box-Cox transformations (MASS package) were applied to normalize values prior to GWAS.

### DNA extraction and genotyping

Genomic DNA from 610 pig liver tissues were extracted using cell lysation with proteinase K (Roth, Karlsruhe, Germany), followed by phenol–chloroform extraction (Sambrook, 2006) with Phase Lock Gel tubes (5 Prime, Hamburg, Germany). The DNA was quantified using a NanoDrop ND-1000 spectrophotometer (Peqlab, Erlangen, Germany). Afterwards, the samples were genotyped using a 60K porcine single nucleotide polymorphisms (SNPs) bead chip (Illumina, San Diego, CA, United States). Data generation was done using GenomeStudio software (version 2.0.3, Illumina) for clustering of genotypes and initial quality control (sample call rate > 95% and SNP call rate > 95%). The missing values were subsequently imputed using fastPHASE [28]. For this imputation, the fastPHASE settings were configured to perform 10 runs of the EM algorithm, with each run consisting of 50 iterations, and single-cluster analysis was conducted with the genotyping error option activated. The bead chip SNP sequences were mapped to the pig genome assembly Sus scrofa 11.1. Markers were further filtered based on Hardy–Weinberg equilibrium, retaining only those loci with P-values exceeding 1 × 10^−6^ and a minor allele frequency (MAF) > 0.05. Additionally, markers on sex chromosomes (X, Y) and markers that did not have clear chromosomal positions were excluded. In total, the number of markers was reduced from 62,549 to 46,800.

### Determination of heritability, genetic correlation, and phenotypic correlation

Heritability of the four traits was examined using the Bayesian statistical trait-specific univariate framework, BGLR (Bayesian Generalized Linear Regression) version 1.1.2 [29]. BGLR was applied to fit a genomic prediction model using a single random effect with an RKHS (Reproducing Kernel Hilbert Space) approach, which captures complex non-linear relationships between genotypes and phenotypes. This method involved computing a genomic relationship matrix (GRM) [30] for the polygenic effect, and performing multiple Markov Chain Monte Carlo (MCMC) chains to estimate heritability for each trait individually. MTM (Multi-Trait Mixed Model) package version 1.0.0 in R was employed to estimate the genetic and phenotypic correlations between traits in bivariate models. For the combined use of BGLR and MTM, default parameters have been used in which the MCMC iteration was set to 200,000 and with a thin interval of 5 to reduce autocorrelation. After completing 200,000 iterations, convergence was assessed by applying the Gelman–Rubin diagnostics to additional Markov chains using the coda package in R (version 0.19–4.1). The potential scale reduction factors (PSRF) were less than 1.1, indicating that the chains had converged successfully [31]. Following the omission of the first 50,000 MCMC iterations as the burn-in phase, the mean genomic heritability ± SD as well as correlation coefficients ± SD were calculated based on the remaining iterations. The data was displayed for each trait pair, providing insights into the magnitude and variability of the phenotypic relationships.

### Genome-wide association study

The processed phenotypic data were integrated with genotype data for genome-wide association studies (GWAS) using the genome association and prediction integrated tool GAPIT version 3 [32] in R v4.1.1. An Enriched Compressed Mixed Linear model (ECMLM) [33], implemented in GAPIT, was used including one principal component (PC) and a kinship matrix using the Zhang approach to account for population stratification and relatedness among individuals [34]. This model extends the standard mixed linear model by compressing individuals into clusters based on genetic similarity, which reduces computational complexity and accounts for genetic relatedness among individuals. The model incorporates both fixed effects such as SNPs and PCs to adjust for population structure and random effects representing the polygenic background. By incorporating both kinship and population structure into the model, the ECMLM effectively controls for confounding factors, enhancing the power to detect true genetic associations with the traits of interest while minimizing false positives [35].

To account for the inter-relatedness of SNP markers, i.e. linkage disequilibrium, SimpleM was employed to estimate the effective number of independent statistical tests [36]. The SimpleM was used in the R environment by analyzing PCs based on a composite linkage disequilibrium matrix from the SNP genotypes. According to the inferred 12,229 independent tests, the suggestive significance threshold in the GWAS has been set at − log_10_(*P*) = 4.09 (1/12,229), and the genome-wide threshold has been set at − log_10_(*P*) = 5.39 (0.05/12,229) [37]. To assess model fit, GAPIT generated quantile–quantile (QQ) plots of observed *P*-values for each trait, comparing them to the expected distribution under the null hypothesis. Manhattan plots were generated for each trait using the ggplot2 package in the R environment to visualize genome-wide association results and, highlight significant markers across the genome.

### Filtering potential candidate genes

Based on the GWAS results, the downstream investigation of candidate genes was implemented by locating the genomic regions using the Ensembl Biomart pig genome (Sscrofa 11.1) within 500 kb upstream and downstream of the significantly associated SNP. Ensembl identifiers and corresponding gene symbols were extracted. The genes that directly contain one of the trait-associated SNPs were taken as positional candidate genes. Additionally, genes with known functional connections to the phenotypes in the respective genomic region were considered functional candidates, based on their functional annotation derived from GeneCards (http://www.genecards.org) [38], and public databases. The Ensembl annotated genes were converted into human orthologous gene symbols using the g:Profiler [39].

## Results

### Phenotypic data analysis

Pigs exhibited significant natural variation in physiological parameters related to maintaining mineral homeostasis and growth. These parameters include blood levels of the vitamin D metabolites calcidiol and calcitriol as well as bone remodelling markers ß-CTX and CICP in the German Landrace pig population as summarized in Table 1. The extensive phenotypic variation highlights the pigs’ individual capacity for resilience in mineral utilization and towards maintaining homeostasis.

**Table 1:** Variation in serum vitamin D metabolites and bone remodeling markers in German Landrace pigs at approximately 160 days of age

Trait	Acronym	Unit	N	Mean	SD	Min	Max
Serum vitamin D_3_ metabolites							
Calcidiol	25(OH)D_3_	ng/ml	608	31.60	3.07	7.82	80.95
Calcitriol	1,25(OH)_2_D_3_	pmol/L	601	224.97	33.54	1.62	734.78
Serum bone markers							
C-terminal telopeptide	β-CTX	ng/ml	602	0.32	0.02	0.04	0.98
C-terminal propeptide of type I collagen	CICP	ng/ml	607	44.92	4.53	17.40	408.01

In the pig population of more than 600 animals, variability was evident in all four traits. The serum levels of calcidiol and calcitriol showed mean values of 31.60 ng/ml and 224.97 pmol/L, respectively. Calcidiol values ranged from 7.82 to 80.95 ng/ml and calcitriol from 1.62 to 734.78 pmol/L. Similarly, the bone remodeling markers ß-CTX and CICP displayed considerable variability, with mean values of 0.32 ng/ml and 44.92 ng/ml respectively, and ß-CTX ranging from 0.04 to 0.98 ng/ml, and CICP varying between 17.4 and 408.01 ng/ml.

### Heritability and correlation analysis

The genomic heritability of the vitamin D hormones and bone remodeling markers revealed moderate to low estimates (Table 2). For serum calcidiol and calcitriol, the heritability was 0.14 and 0.12 respectively. For ß-CTX and CICP, the heritability was 0.15 and 0.12, respectively. The analyses revealed significant phenotypic correlations between calcidiol/ß-CTX and, calcitriol/ß-CTX which suggest a moderate but negative interaction between the first trait pair and moderate positive interaction for the second trait pair (Table 2). Other tested phenotypic correlations were not significant (calcidiol/calcitriol; calcidiol/CICP; calcitriol/CICP, ß-CTX/CICP, suggesting no linear association. A weak but positive genetic correlation was observed between calcidiol/calcitriol (0.202 ± 0.172), and calcidiol/CICP (0.174 ± 0.174), although lack of statistical significance. A significant negative correlation was observed between calcidiol/ß-CTX (-0.350 ± 0.140), whereas calcitriol/ß-CTX (− 0.020 ± 0.208), and ß-CTX/CICP (− 0.012 ± 0.22) had no significant genetic correlation. A weak to moderate negative genetic correlation was observed between calcitriol/CICP (− 0.261 ± 0.206) but calcitriol/ß-CTX − 0.020 ± 0.208) had near zero correlation indicated by their overlapping mean ± SD ranges.

**Table 2:** Estimates of genetic (below the diagonal) and phenotypic (above the diagonal) correlation coefficients with 95% confidence interval (CI) and the genomic heritability (diagonal, bold) for serum concentrations of calcidiol, calcitriol, ß-CTX, and CICP

Trait	Calcidiol	Calcitriol	ß-CTX	CICP
Calcidiol	**0.137 ± 0.029**	0.016 ± 0.044	− 0.121** ± **0.045*	0.008 ± 0.044
Calcitriol	0.202 ± 0.172	**0.123 ± 0.026**	0.106 ± 0.042*	− 0.059 ± 0.042
ß-CTX	− 0.350 ± 0.140*	− 0.020 ± 0.208	**0.153 ± 0.033**	− 0.024 ± 0.042
CICP	0.174 ± 0.174	− 0.261 ± 0.206	− 0.012 ± 0.22	**0.118 ± 0.025**

^*^Significant correlations assessed based on the 95% CI

The highest number of significantly associated SNPs with serum calcidiol were identified on pig chromosomes 2, 8, 9, 12, and 13 (Table 3). In this analysis, 27 SNPs met the significance threshold. On chromosome 2, a large QTL region located between 38.7 to 40.4 Mb was indicated by two SNPs. The SNP, ALGA0013290 (rs81357782) at 39,973,291 bp with a − log_10_(*P*) of 4.96 is situated in an intron of the Neuron navigator 2, *NAV2* gene. Notably, in close proximity, at 45.5 Mb, the SNP marker ALGA0013650 missed the threshold for suggestive evidence with a − log_10_(*P*) of 3.64, but was mapped in *FAR1* and near *PTH*, both of which are interesting functional candidates. Another marker ALGA0016395 at 136,952,778 bp with a − log_10_(*P*) of 7.5 was situated at the upstream region of Calcium-Modulating Cyclophilin Ligand (*CAMLG*) gene. On chromosome 8, in the 67.8 to 70.1 Mb window, a merged QTL region composed of multiple overlapping intervals was highlighted by fifteen markers. Two markers with the highest − log_10_(*P*) were located in the intron of the ADAM Metallopeptidase With Thrombospondin Type 1 Motif 3 (*ADAMTS3*) gene. Another two significant SNPs were identified in proximity to the *GC* gene, which encodes the vitamin D binding protein. A synonymous polymorphism, DIAS0002150 (rs81216908), located at 68,346,526 bp within the GC gene, exhibited a − log_10_(*P*) of 4.54. Additionally, another SNP, ASGA0038904 (rs81401012), was found nearby at 68,532,968 bp. Additionally, six significantly associated SNPs were detected near the albumin (*ALB*) gene, including ASGA0100173 and MARC0109837, both situated in an intronic region of *ALB* at approximately 69,538,176 bp and 69,661,678 bp, respectively. On chromosome 9, one leading SNP, MARC0050167 (rs81240638), was located in the intergenic region at 57,997,009 bp between Sorting Nexin 19 (*SNX19*) and Neurotrimin (*NTM*) genes. On chromosome 12, ALGA0065765 (− log_10_(*P*) = 7.13) mapped in the intron region of the ATP Binding Cassette Subfamily C Member 3 (*ABCC3*).

**Table 3:** Genomic regions derived from SNPs significantly associated with serum calcidiol levels in pigs

Chr	Region in bp	Lead SNP^a^	−log_10_(*P*)	Genes located in the region^b^
2	38,775,035–40,473,291	ALGA0013290	4.96	DBX1, NAV2, PRMT3, SLC6A5
2	136,777,921–137,777,921	MARC0041028	7.5	ENSSSCG00000056520, CATSPER3, **CAMLG**, KCTD16, PITX1, TXNDC15, YIPF5,
8	67,846,526–70,161,678	ASGA0099732	5.03	**ADAMTS3**, **AFP**, AFM, **ALB**, AMCF-2, ANKRD17, COX18, CXCL8, GAS2, **GC**, NPFFR2, PPBP, RASSF6
8	117,758,071–118,758,071	MARC0041943	4.71	MANBA, **NFKB1**, SLC39A8, SLCB1, SLC9B2, UBE2D3
9	57,497,009–58,497,009	MARC0050167	13	ENSSSCG00000045830, ENSSSCG00000057946, SNX19, NTM
12	26,472,131–27,472,131	ALGA0065765	7.13	**ABCC3**
13	130,500,310–131,500,310	MARC0013088	12.59	ATP13A3, CPN2, GP5, **HES1**, LRRC15, OPA1
13	202,722,381–203,722,381	MARC0077025	12.59	DSCAM, GET1, HMGN1, LCA5L, **PCP4**, SH3BGR

^a^Highest significantly associated SNP in the indicated genomic region

^b^The underlined gene symbols indicate a positional candidate, those in bold indicate a functional candidate

In the GWAS data, one SNP (ALGA0107570) at chromosome 6 emerged as the most significantly associated marker with serum calcitriol (Table 4). The region around the SNP contained different genes, with the Podoplanin encoding gene (*PDPN*) as putative functional candidate.

**Table 4:** Genomic regions derived from SNPs significantly associated with serum calcitriol levels in pigs

Chr	Region in bp	Lead SNP^a^	− log_10_(*P*)	Genes located in the region^b^
6	72,346,447–73,346,447	ALGA0107570	4.13	AADACL3, AADACL4, CFAP107, DHRS3, LRRC38, **PDPN**, PRDM2, SNORA59A, VPS13D

^a^Highest significantly associated SNP in the indicated genomic region

^b^The bold gene symbols indicate a functional candidate

Table 5 highlighted the SNPs with significant associations with serum β-CTX levels, identifying genomic intervals on chromosomes 1, 5, 6, and 12. Each of these markers demonstrated strong statistical support, as indicated by − log_10_(*P*) values ranging from 7.5 up to 9.4. Notably, on chromosome 5, the SNP DRGA0006183 (rs81303231) at position 90,817,404 bp was situated within the intron region of the BTG Anti-Proliferation Factor (*BTG1*) gene. On chromosome 6, the SNP ALGA0109178 (rs81337222) at position 10,727 bp fell into a gene-rich region encompassing *ACSF3, ANKRD11,* and *CBFA2T3*. Moving to chromosome 1, the H3GA0053809 (rs80955684) SNP at position 171,665,883 bp was located near *LRFN5*, a gene involved in neurological and cellular signaling with functional implications in skeletal homeostasis. Lastly, on chromosome 12, ALGA0121279 (rs81328842) at position 50,800 bp was surrounded by a diverse array of candidate genes such as *METRNL, FN3K,* and *FASN*.

**Table 5:** Genomic regions derived from SNPs significantly associated with serum ß-CTX levels in pigs

Chr	Region in bp	Lead SNP^a^	−log_10_(*P*)	Genes located in the region^b^
1	171,165,883–172,165,883	H3GA0053809	7.5	ENSSSCG00000055452, ENSSSCG00000054095, ENSSSCG00000056908, LRFN5
5	90,317,404–91,317,404	DRGA0006183	9.4	**BTG1**, ENSSSCG00000060262, ENSSSCG00000051747
6	1–510,727	ALGA0109178	9.4	ACSF3, **ANKRD11**, CBFA2T3, CDH15, CDK10, CDT1, CHMP1A, CTU2, DBNDD1, DEF8, DPEP1, FANCA, GALNS, GAS8, MC1R, PABPN1L, PIEZO1, PRDM7, RNF166, SNAI3, SNORD68, SPATA2L, SPG7, SPIRE2, TRAPPC2L, TUBB3, VPS9D1, ZNF276
12	1–550,800	ALGA0121279	7.5	B3GNTL1, CBR2, CCDC57, CSNK1D, CYBC1, DCXR, DUS1L, ENSSSCG00000055423, **FASN**, FN3K, FN3KRP, **FOXK2**, GPS1, HEXD, LRRC45, METRNL, NARF, OGFOD3, RAC3, RAB40B, RFNG, SLC16A3, TBCD, WDR45B, ZNF750

^a^Highest significantly associated SNP in the indicated genomic region

^b^The underlined gene symbols indicate a positional candidate, those in bold indicate a functional candidate

Table 6 summarizes SNPs significantly associated with serum CICP levels in pigs, pinpointing genomic regions on chromosomes 3, 12, and 16. On chromosome 3, the SNP H3GA0054224 (rs81327306) at 59,308,378 bp was located, harboring several genes i.e. *ATOH8, CAPG*, *ELMOD3*, *GGCX, RETSAT*, *KCMF1*, *TMSB10* within 1 MB around the SNPs. Two SNPs on chromosome 12, ALGA0110696 (rs81339011) at 52,300,193 bp and ASGA0088871 (rs81477907) at 51.21 Mb were surrounded by a particularly dense cluster of candidate genes, with *ALOX15* being the most promising candidate due to functional and positional aspects. Finally, on chromosome 16, ALGA0103470 (rs81330470) at 72,712,730 bp highlights a region containing protein-coding genes *ATPSCKMT*, *CCT5*, *TAS2R1*, and *SEMA5A.*

**Table 6:** Genomic regions derived from SNPs significantly associated with serum CICP levels in pigs

Chr	Region in bp	Lead SNP^a^	− log_10_(*P*)	Genes located in the region^b^
3	58,808,378–59,808,378	H3GA0054224	4.46	**ATOH8**, CAPG, C2orf68, ELMOD3, **GGCX**, GNLY, KCMF1, MAT2A, **RETSAT**, RNF181, SFTPB, ST3GAL5, TMEM150A, TMSB10, USP39, VAMP5, VAMP8
12	51,800,193–52,800,193	ALGA0110696	4.34	ACADVL, ACAP1, ALOX12, **ALOX15**, ARRB2, ASGR1, ASGR2, BCL6B, CAMTA2, C17orf107, C17orf114, C17orf49, CHRNB1, CHRNE, CLEC10A, CLDN7, CTDNEP1, DLG4, DVL2, ELP5, ENO3, **FGF11**, GABARAP, GLTPD2, GPS2, INCA1, KCTD11, KIF1C, MED11, MINK1, NEURL4, PELP1, PHF23, PLSCR3, PLD2, POLR2A, PFN1, RNASEK, RNF167, SENP3, SLC16A11, SLC16A13, SLC2A4, SLC25A11, SPAG7, SPEM2, ssc-mir-195, ssc-mir-324, ssc-mir-497, TM4SF5, TMEM102, TMEM95, TNK1, VMO1, YBX2, ZMYND15, ZNF232
12	50,714,488–51,714,488	ASGA0088871	4.18	AIPL1, C1QBP, DERL2, DHX33, MED31, MIS12, NUP88, PIMREG, PITPNM3, RABEP1, RPAIN, SLC13A5, TXNDC17, WSCD1
16	72,212,730–73,212,730	ALGA0103470	4.09	ATPSCKMT, CCT5, SEMA5A, SNORD123, TAS2R1

^a^Highest significantly associated SNP in the indicated genomic region

^b^The underlined gene symbols indicate a positional candidate, those in bold indicate a functional candidate

## Discussion

This study aimed to elucidate the genetic factors influencing individual variability in mineral utilization by focusing on the vitamin D system and bone remodeling markers in a population of purebred German Landrace pigs. In line with this objective, serum levels of calcidiol and calcitriol were measured in a population of more than 600 pigs, revealing mean values of 31.60 ng/ml and 224.97 pmol/L (93.73 ng/L) respectively. Compared to humans, whose calcidiol levels typically range from 20 to 50 ng/ml with a mean of about 30 ng/ml [40] and calcitriol levels from 29 to 83.6 ng/L, with a mean value of 52.9 ng/L [41], pigs exhibit higher active vitamin D concentrations on average [41]. In adult humans, physiological concentration of serum ß-CTX ranges from 0.56–0.70 ng/ml, which is comparable to observed values in pigs ranging from 0.02–0.98 ng/ml [42]. In healthy adult females, serum CICP levels are approximately 51.5 ng/ml [43] which is close to the observed average levels of 44.92 ng/ml in our study. In fact, a cohort of children demonstrated substantial inter-individual variability in blood CICP levels, highlighting the biological diversity in growth and bone metabolism within populations [44]. The relationship among vitamin D metabolites and bone remodelling markers was evaluated through their heritability, phenotypic and genetic correlations (Table 2). Genomic correlations revealed weak but positive association between calcidiol and calcitriol (0.202 ± 0.172) but no phenotypic correlation (0.016 ± 0.044) was observed between these traits, suggesting that a partly shared genetic influence is masked by environmental or other non-genetic factors at the phenotypic level. A lack of genetic and phenotypic correlation was observed between calcidiol and CICP, which may indicate that bone formation is mainly regulated by factors not directly depending on calcidiol bio-availability. Genetic correlations revealed complex interactions, with calcidiol and β-CTX showing significant negative genetic and phenotypic correlations suggesting potential genetic antagonism between these traits. This finding could indicate higher calcidiol levels are associated with reduced bone resorption, which aligns with the well-established anti-resorptive effects of the vitamin D system on osteoclast activity in humans [45]. However, a statistically significant positive phenotypic correlation was found for calcitriol and β-CTX, which indicate that calcitriol might promote osteoclastogenesis depending on the organism’s Ca balance and developmental stage [9]. Despite the absence of statistical significance, the negative genetic (− 0.261 ± 0.206) and phenotypic correlations (− 0.059 ± 0.042) between calcitriol and CICP suggest that elevated calcitriol levels act in a feedback mechanism with PTH to maintain Ca homeostasis [46, 47]. The absence of a genetic correlation between ß-CTX and CICP (− 0.012 ± 0.22) implies that bone resorption and formation do not share common genetic determinants. These findings emphasize the necessity to carefully tailor dietary vitamin D supplementation and management strategies to the distinct physiological requirements.

### Genomic heritability of vitamin D metabolites and bone markers

The heritability of the vitamin D hormones and bone remodeling markers within the studied pig population indicates a modest genetic influence on these traits. In human studies, the heritability of circulating vitamin D (calcidiol) levels has been shown in different twin studies, with estimations ranging from 16% to as high as > 85% [48, 49]. A study in a domestic Soay sheep population (n = 917) has reported an estimated heritability for plasma vitamin D3 of 0.19 [25]. For calcitriol, the analysis of human twin data indicates a moderate heritability, with estimates from various studies converging on a range of 0.16 to 0.74 [48]. In terms of heritability, the estimates for blood calcidiol and calcitriol align well with studies in humans and sheep populations, which are derived from large cohorts and allow comprehensive insights. For bone remodelling markers, one study demonstrated low heritability of P1NP (procollagen type 1 N-terminal propeptide) and CTX in humans (0.08 and 0.26, respectively) [50]. Another male twin study has shown moderate heritability for PINP (0.29) [51]. A Swedish twin study (n = 102 pairs) revealed a higher heritability estimate (0.58) for serum ß-CTX [52]. Currently, there is no study estimating the heritability of vitamin D metabolites and markers of bone remodelling in pigs, but heritabilities of similar magnitude, i.e. 0.31 and 0.21, have been determined for related traits such as BMD in Danish Yorkshire (n = 537) and Danish Landrace breeds (n = 212), respectively [23]. The moderate heritabilities observed among these traits support efforts for the implementation of new breeding strategies.

### Genomic regions associated with levels of serum vitamin D metabolites

Scientific interest in serum concentration of circulating vitamin D has been long standing owing to their importance in human health including bone integrity and immunity [53]. Existing genome-wide analysis in human studies indicate several functional candidate genes, mainly vitamin D binding protein (*GC*), 7-dehydrocholesterol reductase (*DHCR7*), and cytochrome P-450 family 2R1 (*CYP2R1*) [54]. Although of importance in terms of health and environmental aspects, no genome-wide analysis of vitamin D metabolites has been performed in pigs, i.e., a large animal model with controlled light regimen, feed, and parentage. In the current study, several genomic regions were identified for the association with serum calcidiol by the corresponding leading SNPs pointing to chromosome 2, 8, 9, 12 and 13 with several positional/nearby genes.

In the bloodstream, vitamin D metabolites are predominantly bound to vitamin D-binding protein (VDBP), which is encoded by the *GC* gene and accounts for 85–90% of transport [55]. Albumin (*ALB*) and other lipoproteins transport the remaining 10–15% [56]. Accordingly, various studies demonstrated association between gene variants of VDBP and the blood calcidiol level in humans [15]. In the current study, a 67.8 to 70.1 Mb genomic region on chromosome 8 was found significantly associated with serum calcidiol, where several leading SNPs pointed to candidate genes including *GC* and *ALB.* A synonymous variant (DIAS0002150) at 68.3 Mb has been found in the coding region of the *GC* gene. Two additional SNPs were located in the intron region of the *ALB* gene at 69.5 Mb. Furthermore, in the same genomic region, the Alpha Fetoprotein encoding gene *AFP* emerged as another candidate. It has also been described as a transporter of fatty acids, bilirubin, and steroids [57] suggesting a possible role in vitamin D transportation and cellular delivery.

Associated with serum calcidiol on chromosome 2 at 45.5 Mb, the most functionally relevant candidate genes indicated by ALGA0013650 (rs81358796) are Fatty Acyl-CoA Reductase 1 *FAR1* and Parathyroid hormone *PTH*. Although mammalian FAR1 plays a key role in converting fatty acids into fatty alcohols, this conversion is essential for the biosynthesis of lipids and wax monoesters, integral members of lipid carriers [58]. As vitamin D metabolites are fat soluble hormones, *FAR1* could be involved in setting intracellular microenvironments (i.e., lipid droplets in adipocytes) to impact on vitamin D metabolite levels [59]. Parathyroid hormone (*PTH*) plays a fundamental role in vitamin D activation by stimulating 1α-hydroxylase, the enzyme responsible for converting calcidiol into calcitriol in the kidneys [60].

**Fig. 1:**
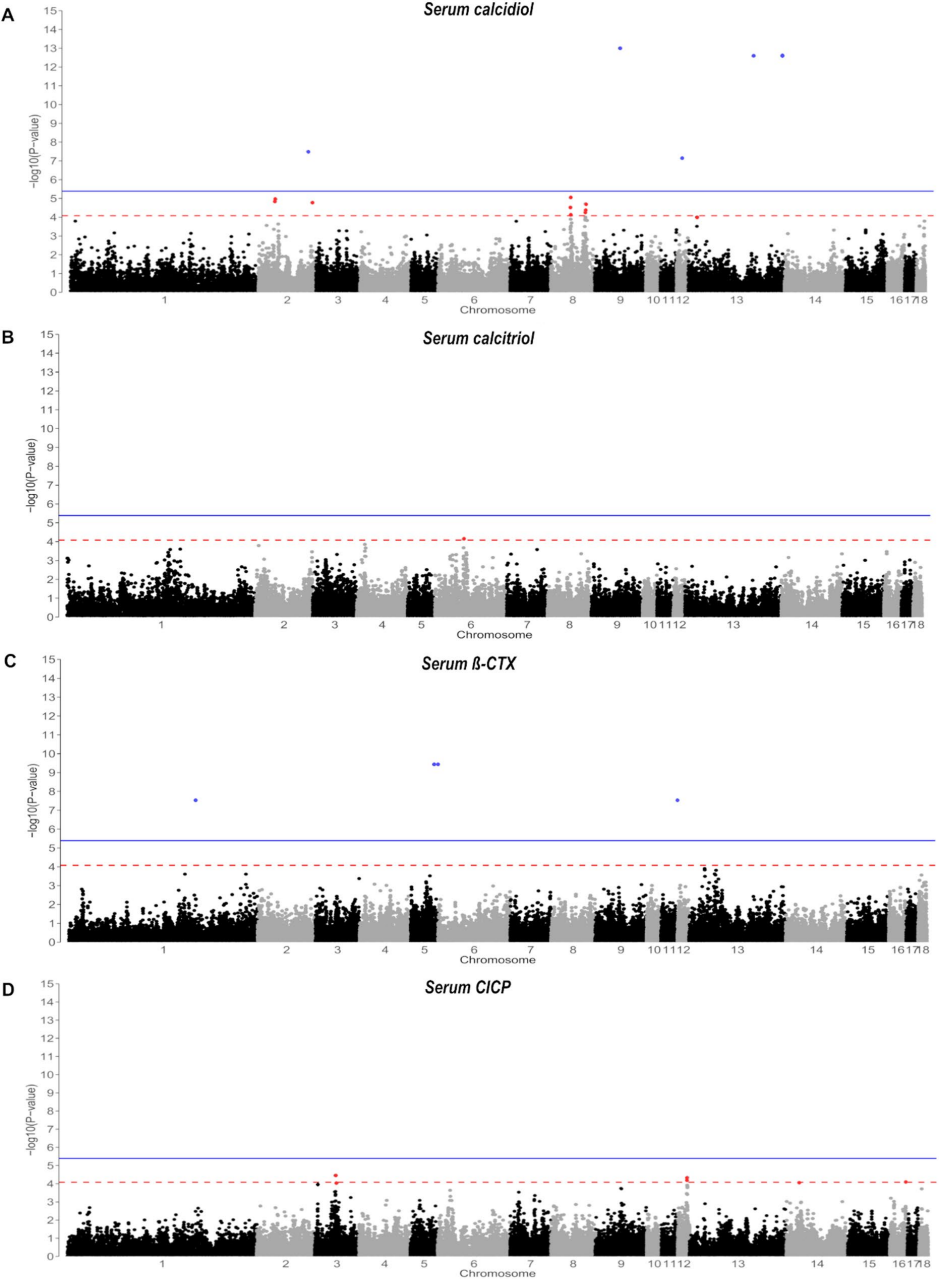
Genome-wide association studies (GWAS) results are visualized using Manhattan plots for serum vitamin D metabolites, i.e. calcidiol (**A**) and calcitriol (**B**), and bone remodelling markers, i.e., ß-CTX (**C**) and CICP (**D**). The statistical significance of single nucleotide polymorphisms (SNPs) is illustrated by the suggestive evidence threshold (red) and genome-wide evidence threshold (blue)

Through its interaction with vitamin D response elements (VDRE), vitamin D plays a crucial role in regulating a wide array of genes and biological processes, potentially affecting nearly 3% of the human genome [61]. Two markers were located on chromosome 8 in the intron region of the Mannosidase Beta encoding gene (*MANBA*), which is in close proximity to the functional candidate gene *NFKB1*, a member of the NF-κB family of transcription factors that play a crucial role in immune response [62]. Given that *NFKB1* is directly regulated by VDRE**,** its association with these SNPs raises the possibility that genetic variation in this region could influence immune-related pathways through vitamin D-dependent transcriptional regulation. The active form of vitamin D also stimulates type 1 procollagen synthesis (*ADAMTS2*) [63] and *ADAMTS3*, which is located in the QTL region of chromosome 8, shares functional similarities with *ADAMTS2* [64]. *ADAMTS3* facilitates the cleavage of procollagen type 1 during the maturation process, an important component of the musculoskeletal tissues [63]. Another marker at 26,972,131 bp on chromosome 12 was located in the intron region of Multidrug Resistance-associated Protein 3 (*ABCC3*) gene, which is a vitamin D receptor (VDR)-regulated gene. This gene is a potential candidate for regulating the transport of bile acids that interacts with vitamin D metabolites [65]. On chromosome 9 and 13, three SNPs (MARC0050167, MARC0013088, MARC0077025) appear to exhibit highest statistical significance (Table 3) in the applied GWAS analysis, while the regions were represented by one single SNPs, respectively (Fig. 1). Although they are located on different chromosomes and/or regions, they exhibit a high linkage disequilibrium with each other, likely due to population genetic effects rather than just being direct causal variants [66]. Accordingly, it is conceivable that they are either misplaced in the genomic assembly or represent artefacts. In fact, MARC0013088 was also classified as suspicious in an earlier study due to potential clustering error [66]. Therefore, further validation is needed to confirm whether these SNPs have any contribution to the observed effect on the blood calcidiol variability.

The analysis of blood calcitriol levels demonstrated substantial individual variability. This variability likely reflects, in part, the reported 3 to 6-h serum half-life of calcitriol in humans [67]. Furthermore, the activity of enzymes responsible for the hydroxylation and dehydroxylation of vitamin D metabolites contributes to this individual variability which is dependent on a variety of environmental factors [6, 68]. The GWAS for serum calcitriol indicated one genomic region on chromosome 6. The lead SNP at 72,846,447 bp is situated in the intergenic region of several Ensembl annotated genes. Of these, Podoplanin (*PDPN)* plays a critical role in osteocytogenesis by facilitating cytoskeletal modifications necessary for dendrite formation in early osteocytes, and its expression increases in response to mechanical load in mice [69].

### Genomic regions associated with serum bone markers level

The GWAS for serum ß-CTX revealed several putative candidate genes across the pig genome. On chromosome 5, a significantly associated SNP is found in the upstream region of the BTG Anti-Proliferation Factor 1 (*BTG1*) gene, which plays an important role in vertebral patterning and axial skeleton development in mice [70]. On chromosome 6, a SNP located in the intergenic region at 10,727 bp, indicated the Ankyrin Repeat Domain 11 (*ANKRD11*) gene, which is a key regulator of intramembranous ossification and craniofacial development. The absence of this gene leads to delayed bone maturation, reduced ossification, and craniofacial abnormalities resembling KBG syndrome, a rare inherited autosomal dominant condition characterized by diverse neurodevelopmental and craniofacial abnormalities. Its role highlights the importance of *ANKRD11* in bone remodeling and midfacial skeletal growth [71]. The genomic region on chromosome 12 harbors Fatty Acid Synthase (*FASN*) gene encoding an enzyme critical for fatty acid biosynthesis in humans [72]. *FASN* is associated with a less favorable bone profile in children, characterized by reduced osteocalcin levels, increased CTX to bone specific alkaline phosphatase (BSAP) ratio, and heightened bone resorption, particularly in the context of low vitamin D levels. Another functional candidate gene in this genomic region is Forkhead Box K2 (*Foxk2*), which promotes adipogenesis by directly enhancing Pparγ1 and Pparγ2 transcription, with its expression upregulated in bone marrow stromal cells during adipogenic stimulation. Given the reciprocal relationship between adipocyte and osteoblast differentiation, *Foxk2* is hypothesized to play a key role in ossification and may serve as a therapeutic target for osteoporosis and bone marrow adipose tissue-related conditions [73].

The GWAS for serum CICP has revealed a number of genes putatively related to bone remodeling. The genomic region on chromosome 3 harbors a number of annotated genes, including *ATOH8, GGCX,* and *RETSAT.* The ATOH8 (Atonal BHLH Transcription Factor 8) is a protein coding gene detected in osteoblasts but not in osteocytes in adult mice [74]. It is a novel bone morphogenic protein (BMP)-target gene in osteoblasts that suppress the transcriptional activity of Runx2, which in turn, reduces the Rankl/Opg expression ratio, and inhibits osteoclastogenesis, ultimately reducing bone loss in mice [74]. The Gamma-Glutamyl Carboxylase (*GGCX*) gene encodes an enzyme called γ-carboxylase which mediates vitamin K dependent carboxylation of osteocalcin (OCN, a hormone secreted by osteoblasts) during post-translational modifications [75]. *GGCX* enhances OCN’s affinity for hydroxyapatite in the bone extracellular matrix (ECM). This modification results in OCN being sequestered in the bone ECM, where it is the most abundant non-collagenous protein [75]. The Retinol Saturase (*RETSAT*) regulates mineral homeostasis, as evidenced by hypercalciuria and hypophosphaturia in RetSat-deficient mice. Its knock-out leads to upregulation of the vitamin D receptor (VDR) and altered expression of its target genes, with RNA-sequencing and GO analysis linking RetSat to cell junction assembly processes in the kidney in a mouse model [76]. Retinoids, such as retinoic acid, are essential regulators of bone remodeling and osteogenesis, influencing the differentiation and activity of osteoblasts and osteoclasts [77]. Therefore, RETSAT plays a crucial role in bone health by modulating the availability of bioactive retinoids that affect bone cell function.

On chromosome 12, one significantly associated SNP was located at 52,300,193 bp at the downstream region of the *ALOX15* gene, encoding 12/15-lipoxygenase, which acts as a negative regulator of peak BMD in mice. Crossbreeding experiments using Alox15 knockout mice confirmed the role of 12/15-lipoxygenase in skeletal development, indicating its influence on bone formation and remodeling processes [78]. A second functional candidate gene in this genomic region is Fibroblast growth factor 11 (*FGF11*), which plays a crucial role in osteoclast function by promoting bone resorption under hypoxic conditions in humans [79. A more comprehensive understanding of the causal relationship between the identified markers and bone remodeling necessitates further investigation into the specific functions of these genes in pigs, as well as the functional characterization of other genes within the associated genomic regions.

## Conclusions

This study represents the first comprehensive analysis of vitamin D metabolites, including serum calcidiol and serum calcitriol, alongside bone remodelling markers such as serum β-CTX and serum CICP in pigs. The moderate heritability estimates for these traits suggest a significant genetic component, while the low correlations among the markers underscore their independence. Through GWAS, several genes—including *PTH*, *GC*, *ALB* for serum calcidiol, *PDPN* for serum calcitriol, *BTG1*, *FASN*, and *FOXK2* for serum ß-CTX, as well as *RETSAT*, *ATOH8*, *FGF11*, *ALOX15* for serum CICP—were identified as promising candidates, deserving further investigation for polymorphisms and direct links to serum parameters. Information can be used to diagnose and predict characteristics of micronutrient utilization, bone development, and a functioning musculoskeletal system.

## Electronic supplementary material

Below is the link to the electronic supplementary material.

Supplementary Material 1

Supplementary Material 2. Figure S1: Quantile-quantile (QQ) plots for serum calcidiol (A), calcitriol (B), ß-CTX (C) and CICP (D)

## Data Availability

The datasets generated and/or analysed during the current study have been deposited in the European Variation Archive (EVA) at EMBL-EBI under accession number PRJEB88145 (https://www.ebi.ac.uk/eva/?eva-study=PRJEB88145).

## Abbreviations

BGLR: Bayesian generalized linear regression
BMD: Bone mineral density
CI: Confidence interval
CICP: Type I C-terminal collagen propeptide
DNA: Deoxyribonucleic acid
ECMLM: Enriched compressed mixed linear model
ELISA: Enzyme linked immunosorbent assay
GAPIT: Genome association and prediction integrated tool
GRM: Genomic relationship matrix
GWAS: Genome-wide association study
MAF: Minor allele frequency
MCMC: Markov chain Monte Carlo
MTM: Multi-trait mixed model
PC: Principal component
PSRF: Potential scale reduction factors
QTL: Quantitative trait loci
RCF: Relative centrifugal force
RKHS: Reproducing Kernel Hilbert space
SD: Standard deviation
SNP: Single nucleotide polymorphism
ß-CTX: C-terminal telopeptide
