# Supplementary figures and images for: Genome-wide association study of blood vitamin D metabolites and bone remodelling markers in pigs
Source: BMC Genomics. 2025 Aug 2;26:718. doi: 10.1186/s12864-025-11914-1 (PMC12318406; doi:10.1186/s12864-025-11914-1)

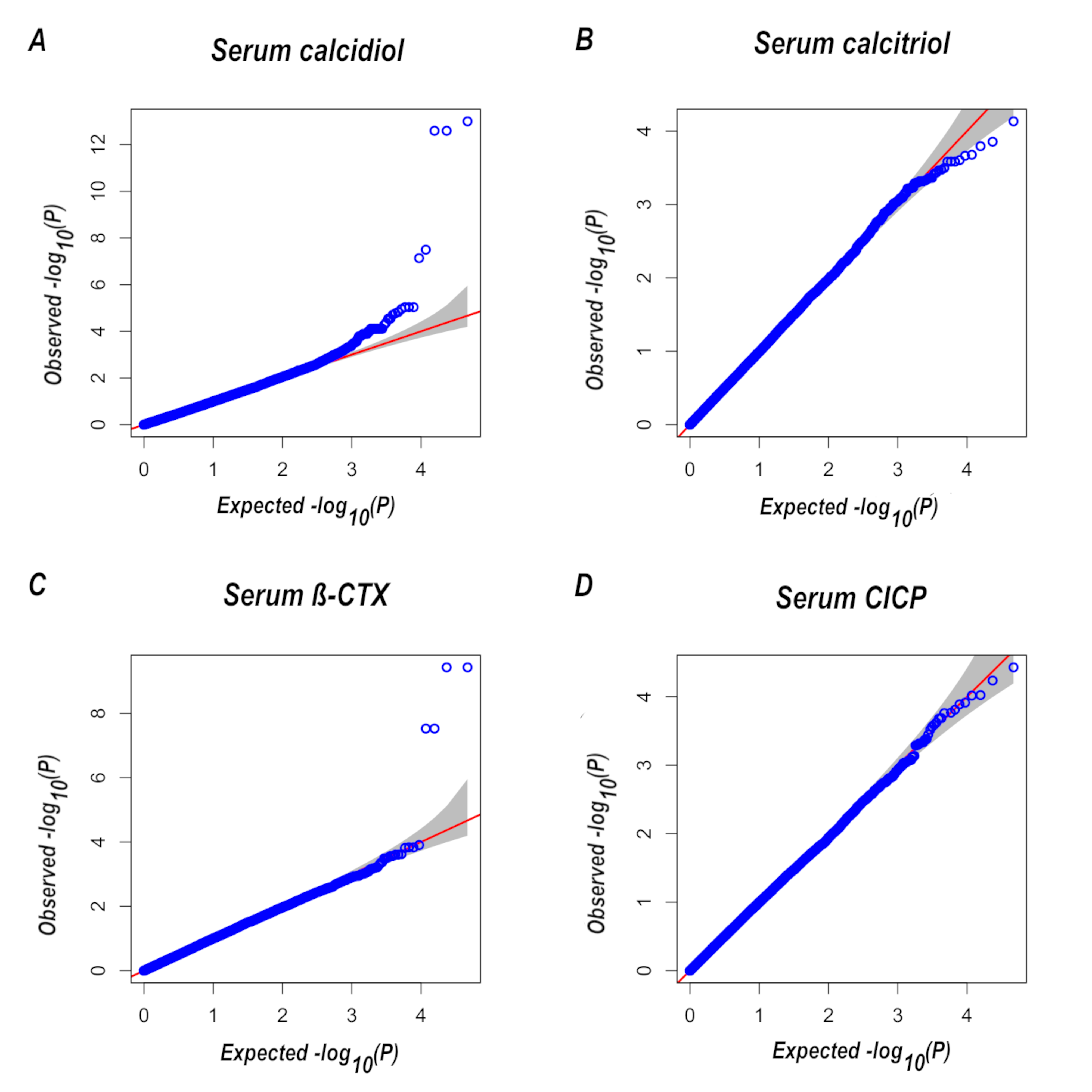

Supplement: Supplementary file 2 — Supplementary Material 2. Figure S1: Quantile-quantile (QQ) plots for serum calcidiol (A), calcitriol (B), ß-CTX (C) and CICP (D) [file 12864_2025_11914_MOESM2_ESM.png]
